# Clinical features, prognostic factors of pediatric acute necrotizing encephalopathy: a 67-case retrospective analysis

**DOI:** 10.3389/fneur.2026.1762541

**Published:** 2026-02-24

**Authors:** Yuyang He, Lanhong Xiang, Qinzhen Cai, Chunhui Yuan, Cong Yao, Shan Huang, Weihong Zhang, Hongmin Zhu

**Affiliations:** 1 Department of Rehabilitation Medicine, Wuhan Children’s Hospital, Tongji Medical College, Huazhong University of Science and Technology, Wuhan, China; 2 School of Medicine, Wuhan University of Science and Technology, Wuhan, China; 3 Department of Laboratory Medicine, Wuhan Children’s Hospital, Tongji Medical College, Huazhong University of Science and Technology, Wuhan, China; 4 Department of Health Care, Wuhan Children’s Hospital, Tongji Medical College, Huazhong University of Science and Technology, Wuhan, China

**Keywords:** acute necrotizing encephalopathy, hormone therapy, influenza virus, prognosis, risk factor

## Abstract

**Background:**

Acute necrotizing encephalopathy is a rapidly and severe encephalopathic condition with no specific treatment, and a generally poor prognosis. This study aimed to investigate the clinical characteristics of pediatric Acute necrotizing encephalopathy, identify key prognostic factors influencing outcomes, and provide evidence to optimize clinical management.

**Methods:**

We retrospectively reviewed the medical records of 67 pediatric ANE patients admitted to Wuhan Children’s Hospital (2014–2022). Baseline demographics, clinical manifestations, laboratory and neuroimaging findings, treatments, and follow-up data were collected. Disease onset was defined as the starting point, and death or 12 months after discharge was set as the study endpoint. Prognostic factors associated with mortality and long-term outcomes were analyzed.

**Results:**

Most patients were aged under 4 years, with prodromal infections were mainly respiratory, with 32.8% associated with influenza virus infection. Eighteen patients died within 3 months, while all 49 survivors exhibited varying degrees of neurological sequelae during 12-month follow-up. Brain magnetic resonance imaging (MRI) commonly shows thalamic lesions, and as the disease progresses, hemorrhage, cystic degeneration, and atrophy may occur. Sixty-three patients received corticosteroids, of whom 21 were treated within 24 h of onset. Univariate logistic regression identified influenza virus infection, prodromal-to-encephalopathy interval ≤24 h, tracheal intubation, Glasgow Coma Scale (GCS) <5, prolonged APTT, and elevated PCT and IL-10 as risk factors for mortality. Multivariate logistic regression demonstrated that hemorrhage and atrophy on follow-up MRI were independent predictors of poor long-term outcome, whereas corticosteroid administration within 24 h of onset was an independent protective factor.

**Conclusion:**

Clinicians should identify influenza-related prodromal infections early (≤24 h), dynamically monitor neuroimaging changes to detect structural brain alterations affecting long-term prognosis, and intervene promptly with glucocorticoid therapy within the 24-h.

## 1 Introduction

Acute necrotizing encephalopathy (ANE) is a fulminant encephalopathy in children, characterized by acute onset, rapid progression, and symmetric necrotic lesions predominantly involving the thalami and brainstem (1). To date, no specific therapeutic regimen has been established for ANE. The mortality rate remains as high as 30%, and the majority of survivors are left with severe neurological sequelae (1, 2), making ANE a major challenge in pediatric neurocritical care worldwide.

In recent years, advances have been made in clinical management (3), with current treatment strategies mainly including immunomodulatory therapy, anti-IL-6 therapy, supportive care, and rehabilitation (4). However, outcome prediction systems remain insufficiently established, and difficulties persist in accurately identifying high-risk patients and tailoring interventions accordingly. Prognostic assessment of ANE is a multidimensional and dynamically evolving process. Previous studies exploring prognostic factors have yielded conflicting results, largely due to variations in follow-up endpoints: some focused on short-term survival and early recovery (5, 6), whereas others investigated the evolution of long-term neurological sequelae (7–9).

Based on this background, we retrospectively analyzed the clinical data of 67 pediatric ANE patients, with a systematic evaluation of their clinical characteristics and neuroimaging evolution. Prognosis was stratified into two dimensions, mortality and long-term outcome, and key determinants for each stage were investigated separately. This study aims to provide evidence for the early identification of high-risk patients and the development of stage-specific, individualized treatment and follow-up strategies, ultimately contributing to improved survival and long-term prognosis of children with ANE.

## 2 Materials and methods

### 2.1 Patients and clinical data

We retrospectively enrolled 67 children diagnosed with ANE at Wuhan Children’s Hospital between January 2014 and June 2022. All patients met the diagnostic criteria for ANE revised by Mizuguchi et al. (10), including: (1) Clinical features: Acute encephalopathy occurring 1–3 days after febrile illness, characterized by rapidly worsening consciousness, (2) Cerebrospinal fluid examination: No significant increase in cell count, elevated protein levels, (3) Imaging findings: Head CT/MRI shows symmetrical, multifocal lesions, necessarily involving bilateral thalami; MRI characteristic manifestations include T2/FLAIR hyperintensity, diffusion restriction on DWI, sometimes accompanied by hemorrhage, (4) Laboratory tests: Serum transaminases elevated to varying degrees, normal blood ammonia. Patients with infectious, metabolic, toxic, or autoimmune encephalopathies were excluded. For death cases, those whose death was unrelated to ANE (e.g., accidental trauma) and cases lost to follow-up were excluded. Clinical data were retrospectively analyzed, including sex, age, season of onset, personal, past medical and family history. Information on prodromal infection symptoms and pathogens, the interval between prodromal infection and encephalopathy onset, and clinical manifestations during the acute stage was collected. We collected laboratory examinations at the first admission, including blood routine tests, liver and renal function, electrolytes, cardiac enzymes, coagulation profile, procalcitonin (PCT), cytokines, and cerebrospinal fluid (CSF) analysis (routine and biochemistry). Glasgow Coma Scale (GCS) is based on the score at the first admission. Neuroimaging data were obtained from cranial MRI, and treatment information was also recorded.

### 2.2 Prognostic evaluation

**Figure 1:**
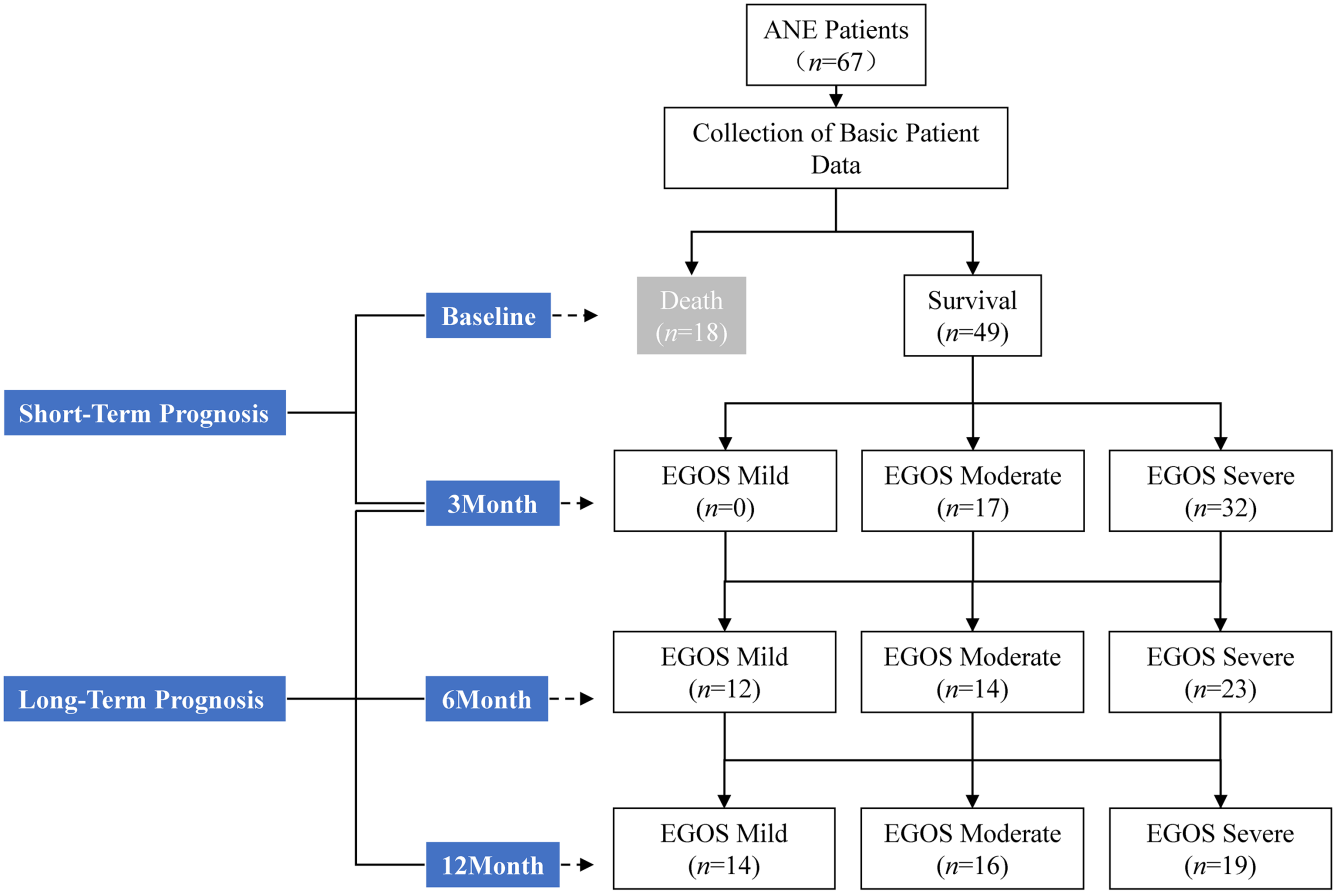
Study flow chart.

To investigate prognostic factors, follow-up was conducted until death for non-survivors and up to 12 months for survivors. Follow-up assessments included the Extended Glasgow Outcome Scale (EGOS) score, residual clinical symptoms, and cranial MRI findings. Outcomes within 3 months were defined as short-term prognosis, whereas outcomes at 12 months were defined as long-term prognosis. Cranial MRI scans were collected at admission and at 1, 3, 6, and 12 months after disease onset. The study flowchart is presented in Figure 1.

Neuroimaging features were defined as follows: hyperintensity on T1-weighted imaging (T1WI) was considered hemorrhage; hypointensity on T1WI, hyperintensity on T2WI, and hypointensity on FLAIR were defined as cystic degeneration; brain atrophy was defined as parenchymal volume loss with or without gliosis and widening of sulci. MRI severity was evaluated using the scoring system proposed by Wong et al. (11), ranging from 0 to 4 points, based on the presence of hemorrhage, cystic changes, brainstem involvement, and white matter involvement (cerebral, cerebellar, or both). Disease severity was further assessed using the ANE Severity Score (ANE-SS) proposed by Yamamoto et al. (12), with a maximum of 9 points, categorized into low risk (0–1), moderate risk (2–4), and high risk (5–9). Prognostic outcomes were assessed using EGOS (13). For survivors, EGOS scores were recorded at 3, 6, and 12 months of follow-up.

### 2.3 Statistical analyses

All statistical analyses were conducted using SPSS version 26.0 (IBM, Armonk, New York, United States), GraphPad Prism version 9.5.1 (GraphPad Software, San Diego, California, United States), and Origin 2021 (OriginLab Corporation, Northampton, MA, United States). The statistical data of clinical variables are presented in the form of proportions and interquartile ranges. Pearson chi square test, Fisher’s exact test, Mann Whitney *U* test, and correlation analysis were used to compare the statistical significance between groups. Logistic regression analysis was performed to determine predictive factors for ANE mortality and long-term prognosis. A Sankey plot of risk factors associated with mortality and long-term outcomes was generated using Origin software. Use Kaplan Meier method to construct survival curves and compare mortality rates among different variable groups. We calculated the odds ratio (OR) and their respective 95% confidence intervals (CI). Statistical tests were two-tailed, and significance was set at *p* < 0.05.

## 3 Results

### 3.1 Patient baseline characteristics

Participants (*n* = 67) with ANE were included in this study. Baseline characteristics are summarized in Table 1. The male-to-female ratio was 1.4:1, with an age range of 1–142 months. Most patients (62.7%) were under 4 years old, and 68.7% had disease onset during the winter or spring. 62 patients (92.5%) had no significant past medical or family history. One patient had an elder brother who developed encephalitis and died at the age of 3 following a similar infection, and genetic testing confirmed a RANBP2 mutation in this patient. Additionally, one patient had a history of motor developmental delay, one had recurrent pneumonia, one had febrile seizures, and one had purulent meningitis.

**Table 1:** Clinical features of 67 pediatric patients with ANE.

**Features**	**Patients (*n*=67)**
**Age, median (IQR) (m)**	39 (22, 76)
≤48	42 (62.7%)
> 48	25 (37.3%)
**Sex**	
Male	39 (58.2%)
Female	28 (41.8%)
**Morbidity season**	
Winter–Spring (December–May)	46 (68.7%)
Summer–Autumn (June–November)	21 (31.3%)
**Symptoms of prodromal infection**	
Fever	67 (100.0%)
Respiratory symptoms	41 (61.2%)
Digestive symptoms	27 (40.3%)
Influenza virus infection	22 (32.8%)
**Intervals from prodromal infection to acute encephalopathy (d)**	
1	24 (35.8%)
2	22 (32.8%)
3	14 (20.9%)
4	5 (7.5%)
5	2 (3.0%)
**Presentation of acute encephalopathy**	
Seizures	55 (82.1%)
Seizures with status epilepticus	17/55 (30.9%)
Seizures without status epilepticus	38/55 (69.1%)
No Seizures	12 (17.9%)
MODS	43 (64.2%)
Shock	8 (11.9%)
DIC	5 (7.5%)
**GCS, median (IQR)**	5 (3,7)
≤8	53 (79.1%)
9-12	11 (16.4%)
≥13	3 (4.5%)
**ANE-SS, median (IQR)**	3 (2,5)
Mild risk	12 (17.9%)
Moderate risk	35 (52.2%)
Severe risk	20 (29.9%)
**Location of lesions**	
Thalamus	67 (100.0%)
Periventricular	44 (65.7%)
Basal ganglia	41 (61.2%)
Brainstem	49 (73.1%)
Cerebral cortex and subcortex	32 (47.8%)
Cerebellum cortex and subcortex	26 (38.8%)
Lesion number	4 ± 1.5
**Radiological findings on follow-Up MRI**	
Hemorrhage	42/67 (62.7%)
Cavitation	31/49 (63.3%)
Atrophy	27/49 (55.1%)
**MRI score, median (IQR)**	3 (2,3)
1	10/49 (20.4%)
2	14/49 (28.6%)
3	14/49 (28.6%)
4	11/49 (22.4%)
**Therapy**	
Admission to ICU	54 (80.6%)
Endotracheal intubation	24 (35.8%)
Methylprednisolone pulse	63 (94.0%)
≤24h	20/63 (31.7%)
≥20mg/kg. d	47/63 (74.6%)
IVIG	60 (89.6%)
Hypothermia	27 (40.3%)
Plasma exchange	1 (1.5%)
Rehabilitation (≥ 6 months hospital rehabilitation)	26/49 (53.1%)
**Prognosis**	
Death	18 (26.9%)
Time of onset to death, d	2-85
Survival	49 (73.1%)
EGOS score (3m), median (IQR)	3 (2, 5)
Mild	0 (0%)
Moderate	17/49 (34.7%)
Severe	32/49 (65.3%)
EGOS score (6m), median (IQR)	5 (3, 6.5)
Mild	12/49 (24.5%)
Moderate	14/49 (28.6)
Severe	23/49 (46.9%)
EGOS score (12m), median (IQR)	5 (3.5, 7)
Mild	14/49 (28.6%)
Moderate	16/49 (32.7%)
Severe	19/49 (38.7%)

m, month; d, day; IQR, interquartile range; MODS, multiple organ dysfunction syndrome; DIC, disseminated intravascular coagulation; ICU, intensive care unit; GCS, Glasgow Coma Scale; ANE-SS, ANE Severity Score; MRI, magnetic resonance imaging; IVIG, intravenous immune globulin.; EGOS, Extended Glasgow Outcome Scale.

All patients presented with fever, 61.2% developed respiratory symptoms, and 27% exhibited gastrointestinal symptoms. Laboratory testing confirmed influenza virus infection in 32.8% of cases. In this cohort, acute encephalopathy symptoms appeared 1–5 days after the prodromal infection, with 35.8% of children exhibiting encephalopathy symptoms within one day. Neurological manifestations included impaired consciousness (GCS ≤8) in 79.1% of patients and seizures in 82.1%, of whom 30.9% experienced status epilepticus. Multiple organ dysfunction syndrome (MODS) was observed in 64.2% of patients, while shock and disseminated intravascular coagulation (DIC) occurred in 11.9 and 7.5%, respectively.

Of the 67 patients, 18 (26.9%) died, with time to death ranging from 2 to 85 days (mean, 21 days). Thirteen of these deaths occurred within the first month. Among the 49 survivors, EGOS scores at 3, 6, and 12 months of follow-up are shown in Figure 1.

### 3.2 Laboratory findings and ANE-SS scores on admission

All patients underwent baseline laboratory testing on admission, including complete blood count, liver and renal function, electrolytes, cardiac enzymes, and coagulation profile. Abnormalities were observed in activated partial thromboplastin time (APTT), aspartate aminotransferase (AST), lactate dehydrogenase (LDH), procalcitonin (PCT), interleukin-6 (IL-6), and interleukin-10 (IL-10) (Table 2). Median APTT was 39.00 (29.00–46.10) s, with a maximum of 89 s; AST was 121.00 (48.00–292.00) U/L, peaking at 3732 U/L; LDH was 461.00 (320.00–654.00) U/L, with a maximum of 9,082 U/L; and PCT was 1.32 (0.33–15.13) ng/mL, reaching up to 102.21 ng/mL. Among the 42 patients tested for serum cytokines, median IL-6 was 15.97 (5.67–84.46) pg/mL, and IL-10 was 8.12 (3.58–27.92) pg/mL. The ANE Severity Score (ANE-SS) ranged from 0 to 8. 17.9% of patients were categorized as low risk, 52.2% as moderate risk, and 29.9% as high risk based on risk stratification.

**Table 2:** Laboratory indicators at first admission of 67 pediatric patients with ANE.

**Laboratory findings**	**Median (IQR)**
PLT (×10^9^/L)	192.00 (120.00, 280.00)
APTT (s)	39.00 (29.00, 46.10)
ALT (U/L)	67.00 (37.00, 150.00)
AST (U/L)	121.00 (48.00, 292.00)
LDH (U/L)	461.00 (320.00, 654.00)
CK (U/L)	290.00 (86.00, 778.00)
PCT (ng/mL)	1.32 (0.33, 15.13)
CSF protein (g/L)	0.75 (0.50, 1.39)
CSF WBC	3.00 (2.00, 5.50)
IL-2 (pg/mL)	2.45 (2.06, 3.52)
IL-4 (pg/mL)	2.25 (1.72, 3.39)
IL-6 (pg/mL)	15.97 (5.67, 84.46)
IL-10 (pg/mL)	8.12 (3.58, 27.92)
TNF-α (pg/mL)	2.28 (1.78, 2.97)
IFN-γ (pg/mL)	2.75 (1.60, 5.08)

IQR, interquartile range; PLT, platelet count; ALT, alanine aminotransferase; AST, aspartate transaminase; LDH, lactase dehydrogenase; CK, creatine kinase; APTT, activated partial thromboplastin time; CSF, cerebrospinal fluid; PCT, procalcitonin; IL, interleukin; TNF, tumor necrosis factor; IFN, interferon.

### 3.3 Characteristic changes on brain MRI

All patients demonstrated thalamic involvement on acute-phase cranial MRI, with varying degrees of additional involvement in other regions (Table 1). On diffusion-weighted imaging (DWI), 82.1% showed a central hypointense zone in the bilateral thalami, surrounded by a hyperintense ring, with an additional outer hypointense rim; on apparent diffusion coefficient (ADC) maps, this corresponded to a central hyperintensity surrounded by hypointensity and an outer hyperintensity, forming a “tricolor pattern.” In 17.9% of patients, DWI revealed a central thalamic hyperintensity with peripheral hypointensity, with reversed features on ADC maps, consistent with a “bicolor pattern.”

MRI follow-up was available for 49 survivors for ≥12 months (Table 3). Serial imaging demonstrated dynamic disease evolution, with hemorrhage observed in 62.7% (42/67), cystic degeneration in 63.3% (31/49), and brain atrophy in 55.1% (27/49). Based on the MRI scoring system proposed by Wong in 2006, scores among patients with ≥12 months of follow-up were distributed as follows: 20.4% scored 1, 28.6% scored 2, 28.6% scored 3, and 22.4% scored 4.

**Table 3:** Univariate comparison of clinical features and short-term prognosis in ANE patients.

**Features**	**Death group (*n*=18)**	**Survival group (*n*=49)**	***P* value**
Age, median (IQR) (m)	42.5(23.75,81.25)	38.4(16.25,72.50)	0.392
> 48	7(38.9%)	18(36.7%)	0.872
≤ 48	11(61.1%)	31(63.3%)	
Sex			
Female	10(55.6%)	18(36.7%)	0.166
Male	8(44.4%)	31(63.3%)	
**Symptoms of prodromal infection**			
Fever	18(100%)	49(100%)	—
Respiratory symptoms	14(77.8%)	27(55.1%)	0.091
Digestive symptoms	8(44.4%)	19(38.8%)	0.675
Influenza virus infection	11(61.1%)	11(22.4%)	0.003
**Intervals from prodromal infection to acute encephalopathy (h)**			
>24	7(38.9%)	36(73.5%)	0.009
≤24	11(61.1%)	13(26.5%)	
Time of onset to death, median (IQR) (d)	11(4.75-33)	—	—
**Presentation of acute encephalopathy**			
Seizures			
No	3(16.7%)	9(18.4%)	1.000
Yes	15(83.3%)	40(81.6%)	
Seizures with status epilepticus	6(33.3%)	11(22.4%)	—
Seizures without status epilepticus	9(50.0%)	29(59.2%)	—
GCS, median (IQR)	3(3-3.5)	6(5-9)	<0.001
≥5	4(22.2%)	40(81.6%)	<0.001
<5	14(77.8%)	9(18.4%)	
ANE-SS, median (IQR)	4.5(3,5)	3(2,4)	0.006
Mild risk	0	12(24.5%)	0.015
Moderate risk	9(50.0%)	26(53.1%)	
Severe risk	9(50.0%)	11(22.4%)	
MODS	12(66.7%)	31(63.3%)	0.797
Shock	7(38.9%)	1(2.0%)	<0.001
DIC	3(16.7%)	2(4.1%)	0.116
**Location of lesions**			
Thalamus	18(100%)	49(100%)	—
Periventricular	12(66.7%)	32(65.3%)	0.917
Basal ganglia	14(77.8%)	27(55.1%)	0.091
Brainstem	16(88.9%)	33(67.3%)	0.120
Cerebral cortex and subcortex	10(55.6%)	22(44.9%)	0.439
Cerebellum cortex and subcortex	10(55.6%)	16(32.7%)	0.088
**Therapy**			
Endotracheal intubation	12(66.7%)	12(24.5%)	0.001
Methylprednisolone pulse	17(94.4%)	46(93.9%)	1.000
≤24h	2(11.8%)	18(39.1%)	0.038
≥20mg/kg. d	12(70.6%)	35(76.1%)	0.747
IVIG	18(100%)	42(85.7%)	0.176
Hypothermia	6(33.3%)	21(42.9%)	0.580
Plasma exchange	1(5.6%)	0	0.269

m, month; d, day; IQR, interquartile range; MODS, multiple organ dysfunction syndrome; DIC, disseminated intravascular coagulation; GCS, Glasgow Coma Scale; ANE-SS, ANE Severity Score; IVIG, intravenous immune globulin.

### 3.4 Treatment status

Most patients (80.6%) were admitted to the pediatric intensive care unit (PICU), with a median PICU stay of 7 (4–12) days. Tracheal intubation was required in 35.8% of cases. Corticosteroids were administered in 94% of patients, with 31.7% receiving steroids within 24 h of onset. High-dose methylprednisolone therapy (≥20 mg/kg/day) was used in 74.6% of cases. Intravenous immunoglobulin (IVIG) was administered in 89.6%, hypothermia therapy in 40.3%, and plasma exchange in 1.5%. Among survivors, 38.8% underwent ≥6 months of standardized rehabilitation therapy at specialized hospitals (Table 1).

### 3.5 Mortality risk factors

**Figure 2:**
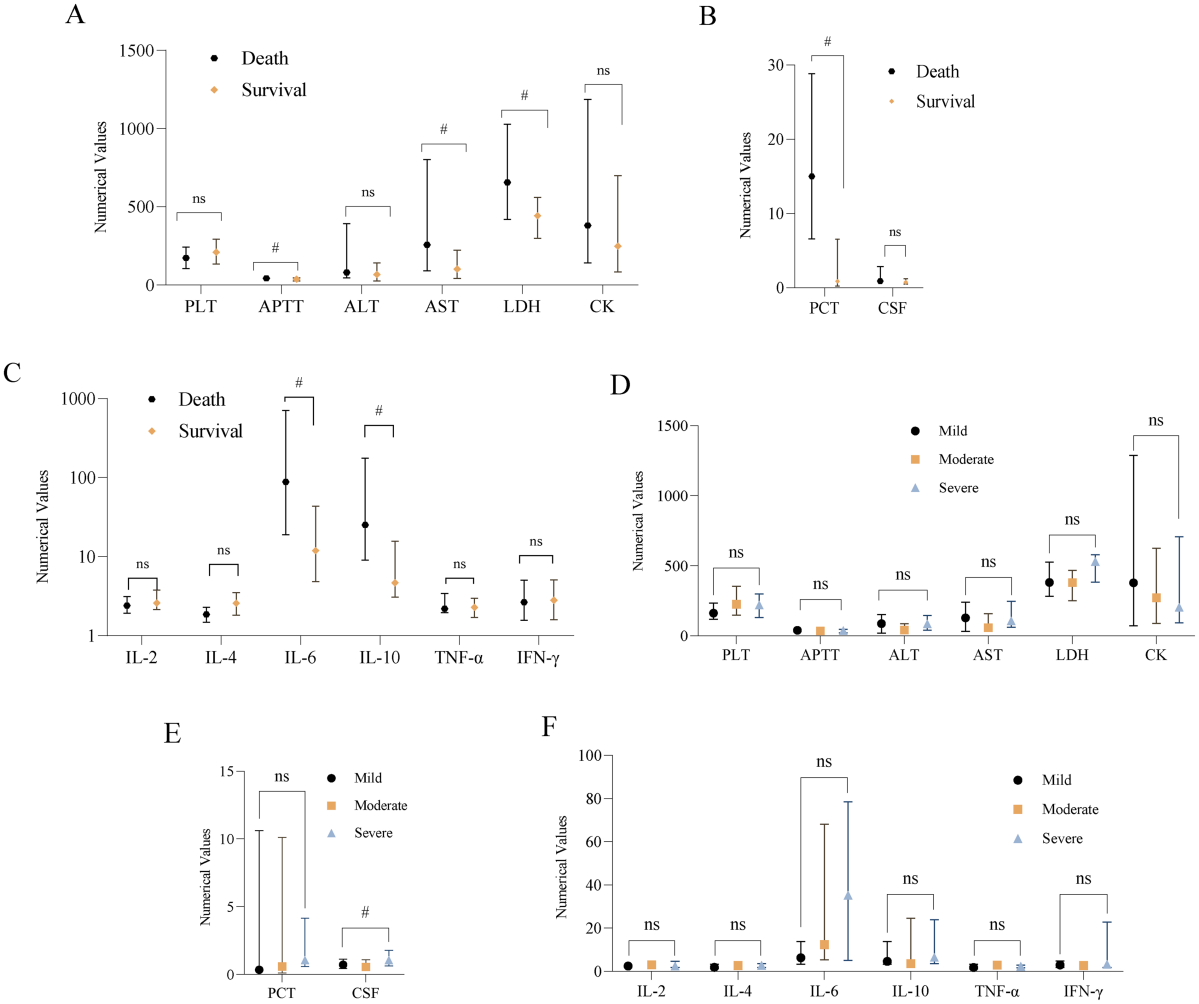
Main biochemical indicators box plot. **(A–C)** Short-term prognostic box plot of distribution comparison of main biochemical indicators. **(D–F)** Long-term prognostic box plot of distribution comparison of main biochemical indicators. ns, no significant; CSF, cerebrospinal fluid, ^#^*p <* 0.05.

Analysis of the association between clinical features and mortality in children with ANE showed that influenza virus infection, an interval of ≤24 h from prodromal infection to onset of encephalopathy, GCS score <5, shock during the acute phase, and the need for tracheal intubation were significantly associated with increased risk of death. In contrast, administration of corticosteroids within 24 h of onset was associated with improved survival (Table 3). Laboratory analyses indicated that death patients were more likely to present with prolonged APTT and elevated levels of AST, LDH, PCT, IL-6, and IL-10 (Figures 2A–C).

To explore the related factors of ANE mortality risk, we used univariate logistic regression analysis. The results identified influenza virus infection, prodromal infection to encephalopathy interval ≤24 h, GCS score <5, acute-phase shock, need for tracheal intubation, higher ANE-SS score, prolonged APTT, and elevated PCT and IL-10 as significant risk factors for short-term mortality (Table 4).

**Table 4:** Univariate logistic regression analysis of factors related to short-term prognosis in ANE patients.

**Features**	**β**	**OR(95% CI)**	***P* value**
Influenza virus infection	1.692	5.429 (1.700,17.337)	0.004
Intervals from prodromal infection to acute encephalopathy (h)	1.471	4.352 (1.391,13.610)	0.011
GCS Classification	2.744	15.556 (4.132,58.567)	<0.001
Shock	3.419	30.545 (3.400,274.390)	0.002
Endotracheal intubation	1.819	6.167 (1.901,20.000)	0.002
Time points for hormone therapy	-1.573	0.207 (0.042 ~ 1.017)	0.052
ANE-SS	0.539	1.715 (1.162,2.530)	0.007
APTT (s)	0.062	1.064 (1.009,1.122)	0.023
AST (U/L)	0.000	1.000 (1.000,1.001)	0.310
LDH (U/L)	0.000	1.000 (1.000,1.001)	0.164
PCT (ng/ml)	0.050	1.051 (1.011,1.093)	0.012
IL-6	0.005	1.005 (0.998,1.013)	0.128
IL-10	0.025	1.025 (1.002,1.050)	0.036

GCS, Glasgow Coma Scale; APTT, activated partial thromboplastin time; AST, aspartate transaminase; LDH, lactase dehydrogenase; PCT, procalcitonin; IL, interleukin; *β*, coefficient; OR, odds ratio; CI, confidence interval.

**Figure 3:**
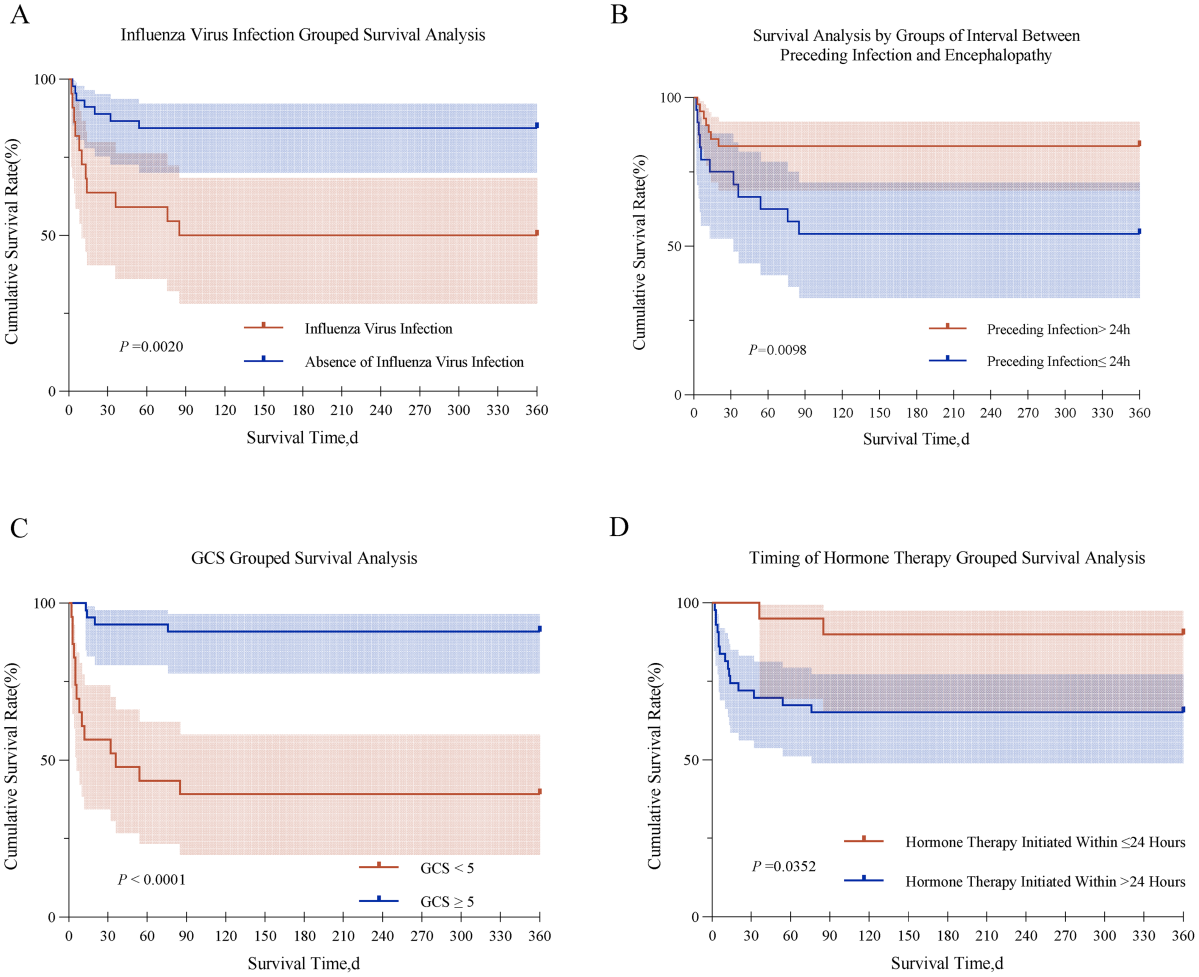
Kaplan–Meier curve for partial death-related risk factors of ANE. **(A)** Influenza Virus Infection Grouped Survival Analysis. **(B)** Survival Analysis by Groups of Interval. Between Preceding Infection and Encephalopathy. **(C)** GCS Grouped Survival Analysis. **(D)** Timing of Hormone Therapy Grouped Survival Analysis.

Kaplan–Meier survival curves were plotted to examine mortality across different risk factors (Figure 3). Compared with non-infected patients, those with influenza virus infection had a higher mortality rate (11/22 vs. 7/45, 50.0% vs. 15.6%). Patients with prodromal infection to encephalopathy interval ≤24 h had higher mortality than those with >24 h (11/24 vs. 7/43, 45.8% vs. 16.3%). Mortality was markedly higher in patients with GCS <5 (14/23 vs. 4/44, 60.9% vs. 9.1%). Conversely, corticosteroid treatment within 24 h of onset reduced mortality (2/20 vs. 15/43, 10.0% vs. 34.9%).

**Figure 4:**
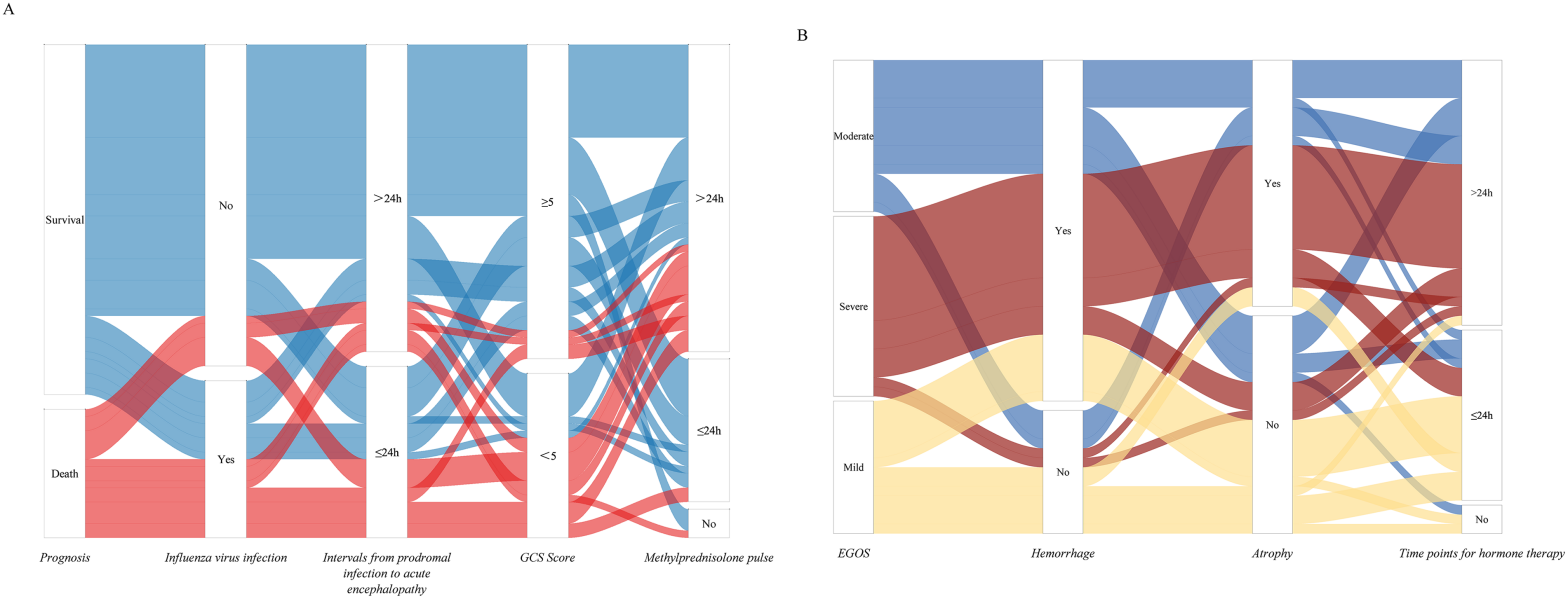
Alluvial plot of changes of ANE. **(A)** Alluvial plot of changes in partial death factors of ANE. **(B)** Alluvial plot of changes in long-term prognostic factors of ANE.

To visually represent these findings, a Sankey diagram was generated showing viral infection, prodromal-to-encephalopathy interval, GCS score, corticosteroid administration timing, and survival status (Figure 4A). These results suggest that children with ANE who have influenza virus infection, prodromal-to-encephalopathy interval ≤24 h, or GCS <5 should be closely monitored for mortality risk, and corticosteroid therapy should be initiated within 24 h whenever possible.

### 3.6 Influencing factors of long-term prognosis

Analysis of clinical features and long-term outcomes revealed that GCS <5, the presence of hemorrhage, cystic degeneration, or atrophy on follow-up brain MRI, higher Wong 2006 MRI scores, and elevated cerebrospinal fluid protein levels were associated with worse EGOS scores at long-term follow-up. Conversely, corticosteroid administration within 24 h of onset and ≥6 months of standardized hospital-based rehabilitation were associated with better long-term outcomes and lower EGOS scores (Table 5 and Figures 2D–F).

**Table 5:** Univariate logistic regression analysis of factors related to long-term prognosis in ANE patients.

**EGOS Classification**	**Mild group (*n*=14)**	**Moderate group (*n*=16)**	**Severe group (*n*=19)**	***P* value**
Age, median (IQR) (m)	63.00 (32.00, 84.25)	36.50(18.50,73.00)	27.00(13.20,42.00)	0.177
>48	8(57.1%)	6(37.5%)	4(21.1%)	0.104
≤48	6(42.9%)	10(62.5%)	15(78.9%)	
Sex				
Female	3(21.4%)	8(50.0%)	7(36.8%)	0.269
Male	11(78.6%)	8(50.0%)	12(63.2%)	
**Symptoms of prodromal infection**				
Fever	14(100.0%)	16(100.0%)	19(100.0%)	—
Respiratory symptoms	9(64.3%)	9(56.3%)	9(47.4%)	0.623
Digestive symptoms	5(35.7%)	7(43.8%)	7(36.8%)	0.882
Influenza virus infection	4(28.6%)	4(25.0%)	3(15.8%)	0.655
**Intervals from prodromal infection to acute encephalopathy (h)**				
>24	10(71.4%)	13(81.3%)	13(68.4%)	0.679
≤24	4(28.6%)	3(18.8%)	6(31.6%)	
**Presentation of acute encephalopathy**				
GCS, median (IQR)	7(5.75,9.25)	6(6,9)	5(3,6)	0.006
≥5	13(92.9%)	15(93.8%)	12(63.2%)	0.043
<5	1(7.1%)	1(6.3%)	7(36.8%)	
ANE-SS, median (IQR)	3.00(2.00,5.00)	3.00(1.25,4.00)	3.00(1.00,4.00)	0.619
Mild risk	1(7.1%)	4(25.0%)	7(36.8%)	0.397
Moderate risk	9(64.3%)	9(56.3%)	8(42.1%)	
Severe risk	4(28.6%)	3(18.8%)	4(21.1%)	
MODS	6(42.9%)	10(62.5%)	15(78.9%)	0.104
Shock	0	0	1(5.3%)	1.000
DIC	0	1(6.3%)	1(5.3%)	1.000
**Location of lesions**				
Thalamus	14(100.0%)	16(100.0%)	19(100.0%)	—
Periventricular	7(50%)	10(62.5%)	15(78.9%)	0.216
Basal ganglia	6(42.9%)	8(50.0%)	13(68.4%)	0.304
Brainstem	10(71.4%)	12(75.0%)	11(57.9%)	0.554
Cerebral cortex and subcortex	6(42.9%)	5(31.3%)	11(57.9%)	0.283
Cerebellum cortex and subcortex	3(21.4%)	5(31.3%)	8(42.1%)	0.473
Hemorrhage	7(50.0%)	12(75.0%)	17(89.5%)	0.041
Cavitation	3(21.4%)	12(75.0%)	14(73.7%)	0.003
Atrophy	2(14.3%)	9(56.3%)	15(78.9%)	0.001
MRI score, median (IQR) (m)	2(1,3)	2.5(2,3)	3(2,4)	0.014
**Therapy**				
Endotracheal intubation	2(14.3%)	2(12.5%)	8(42.1%)	0.094
Methylprednisolone pulse	12(85.7%)	15(93.8%)	19(100.0%)	0.182
≤24h	11/12(91.7%)	4/15(26.7%)	3/19(15.8%)	<0.001
≥20mg/kg. d	10/12(83.3%)	12/15(80.0%)	13/19(68.4%)	0.682
IVIG	12(85.7%)	13(81.3%)	17(89.5%)	0.877
Hypothermia	5(35.7%)	8(50.0%)	8(42.1%)	0.73
Plasma exchange	0	0	0	—
Rehabilitation	8(57.1%)	12(75.0%)	6(31.6%)	0.035

m, month; d, day; IQR, interquartile range; MODS, multiple organ dysfunction syndrome; DIC, disseminated intravascular coagulation; GCS, Glasgow Coma Scale; IVIG, intravenous immune globulin.

To identify independent predictors of long-term prognosis, univariate and multivariate logistic regression analyses were performed. The results demonstrated that hemorrhage and brain atrophy on follow-up MRI were independent risk factors for poor long-term outcomes, whereas corticosteroid therapy within 24 h of onset was an independent protective factor (Table 6). A Sankey diagram illustrating the distribution of long-term prognostic factors among 49 children showed that patients with milder EGOS scores during follow-up rarely had hemorrhage (7/14, 50%) or atrophy (12/14, 85.7%), and most received early corticosteroid treatment within 24 h (11/14, 78.6%) (Figure 4B).

**Table 6:** Univariate and multivariate ordinal logistic regression analyses of long-term prognosis in ANE patients.

**Features**	**β**	**OR (95% CI)**	***P* value**	**β**	**OR (95% CI)**	***P* value**
GCS Classification	1.995	7.352(1.348, 40.103)	0.021	0.853	2.348(0.337, 16.374)	0.389
Hemorrhage	1.582	4.863 (1.385, 17.072)	0.014	2.016	7.509(1.398, 40.328)	0.019
Cavitation	1.660	5.258 (1.645, 16.807)	0.005	0.332	1.393(0.308, 6.313)	0.667
Atrophy	2.154	8.619 (2.586, 28.724)	0.000	2.030	7.611(1.493, 38.792)	0.015
Time points for hormone therapy	-2.683	0.068 (0.331, 1.421)	0.000	-2.338	0.096(0.022, 0.420)	0.002
Rehabilitation	0.954	2.597(0.882, 7.647)	0.083			
CSF protein (g/L)	0.806	2.239 (0.920, 5.448)	0.076			

GCS, Glasgow Coma Scale; *β*, coefficient; OR, odds ratio; CI, confidence interval.

## 4 Discussion

Since its first report in 1995 (14), ANE has been recognized as a sporadic, severe pediatric encephalopathy worldwide, with no significant racial predilection and affecting children across all age groups. Recurrent or familial cases (ANE1), associated with RANBP2 gene mutations, have also been documented. In this study, ANE predominantly affected children under 4 years of age, with higher incidence in winter and spring, and respiratory infections were the main prodromal symptom; 32.8% of patients had concomitant influenza virus infection. ANE is characterized by a rapidly progressive clinical course, particularly in cases with influenza virus infection or a prodromal-to-encephalopathy interval ≤24 h, which were identified as high-risk factors for mortality, suggesting that influenza may be a key trigger (7, 15). All patients exhibited thalamic lesions on brain MRI, with the typical ADC “tricolor/bicolor” pattern (16). Moreover, early corticosteroid administration (≤24 h from onset) was closely associated with both reduced mortality and improved long-term outcomes. Clinicians should therefore maintain a high index of suspicion for ANE during influenza season, promptly perform MRI for diagnosis, and initiate early corticosteroid therapy. Additionally, influenza vaccination may reduce the risk of ANE (17), and should be encouraged in children without contraindications.

Regarding short-term mortality, influenza virus infection, prodromal-to- encephalopathy interval ≤24 h, shock, and low GCS score were identified as key factors in ANE-related deaths. Therefore, in clinical practice, when a child has an influenza virus infection, rapid disease progression, shock, or a low GCS score, the child is at high risk of death and immediate treatment measures are required. Viral encephalitis typically affects superficial brain regions, is usually controllable, and most patients recover with standard treatment. In contrast, ANE induced by viral infection triggers systemic inflammation, disrupts the blood–brain barrier, and causes deep gray matter necrosis (18), often associated with a cytokine storm following post-infectious immune dysregulation (19), elevated IL-6 and IL-10 levels (20, 21), and abnormal cardiac, hepatic, and infection markers (22, 23). In our cohort, death patients exhibited significantly higher IL-6, AST, LDH, PCT, and IL-10 at admission compared with survivors, further supporting the role of inflammatory storm and secondary multi-organ injury in ANE mortality. Consequently, early assessment of systemic inflammation and multi-organ involvement, along with timely anti-inflammatory intervention and intensive monitoring, is warranted for patients with prodromal influenza infection and a prodromal-to-encephalopathy interval ≤24 h to reduce mortality risk.

For long-term prognosis, hemorrhage and atrophy on follow-up MRI were independent risk factors, indicating that irreversible brain injury is central to persistent neurological deficits. Higher Wong MRI scores (reflecting multi-regional involvement) were also associated with poor outcomes, suggesting that neuroimaging can serve as an objective indicator for long-term prognosis. Early corticosteroid therapy within 24 h of onset emerged as an independent protective factor, likely due to suppression of the inflammatory response and preservation of blood–brain barrier integrity, thereby mitigating structural brain damage, consistent with previous studies advocating early immunomodulatory intervention to improve outcomes (24). Neuroimaging studies show that ANE lesions often involve symmetrically distributed deep brain regions with diffuse involvement (25). In our cohort, MRI lesions evolved dynamically over the disease course (hemorrhage → cystic degeneration → atrophy), mirroring the pathological process (edema → necrosis → repair). Thus, regular follow-up brain MRI facilitates objective assessment of cerebral functional status and adjustment of rehabilitation strategies to mitigate functional deficits caused by structural brain damage.

Currently, ANE treatment is primarily supportive. In this study, corticosteroids, IVIG, and mild hypothermia were the main interventions. IVIG showed no significant association with outcomes, consistent with previous reports (26, 27). However, early methylprednisolone administration (within 24 h) significantly improved survival and long-term outcomes. Although the optimal timing and dosing of corticosteroids remain debated (28–30), our study found no difference in prognosis between high-dose (≥20 mg/kg/day) and lower-dose (<20 mg/kg/day) methylprednisolone. Nevertheless, initiation within 24 h was strongly associated with reduced mortality and served as an independent protective factor for long-term outcomes. Therefore, clinicians are advised to administer corticosteroids as early as possible (within 24 h) to reduce inflammation, protect neurons, and improve prognosis (26, 27).

This study included 67 ANE patients, representing a larger cohort than previous studies (1), allowing for a more comprehensive characterization of clinical features and disease course, enhancing the reliability and representativeness of our findings. Based on the disease’s clinical trajectory, early corticosteroid therapy (≤24 h) is particularly crucial for patients with viral infection and a prodromal-to- encephalopathy interval ≤24 h. Long-term prognosis requires close monitoring of neurological recovery, including follow-up MRI for hemorrhage or atrophy, to understand dynamic changes in disease progression and prognostic factors. All patients exhibited thalamic involvement, which may lead to epilepsy, cognitive, language, and motor impairments (31); highlighting the importance of early rehabilitation once the acute phase stabilizes. While multivariate logistic regression did not reveal a significant association between rehabilitation and EGOS scores, univariate analysis suggested that ≥6 months of hospital-based rehabilitation had a positive impact. We considered factors that might be related to rehabilitation outcomes, such as the possibility that patients who improved after rehabilitation may have longer stays than those with severe impairments. However, we still recommend early, continuous, and structured rehabilitation, which may help improve long-term neurological outcomes.

Several limitations should be acknowledged. First, although the study included 67 patients, the sample may still be insufficient to fully elucidate the association between clinical characteristics and prognosis, such as the impact of corticosteroid dose on mortality and long-term outcomes. Second, this single-center study may limit the generalizability of findings and the comprehensiveness of clinical phenotypes. Third, although hemorrhage and atrophy on follow-up MRI were identified as independent predictors of long-term prognosis, lesion size was not quantitatively analyzed. Future studies should expand sample size, perform quantitative neuroimaging analyses, and examine dynamic correlations with neurological function and quality of life to better inform long-term management. Finally, the follow-up period was 12 months, which may not capture more distal neurological sequelae (e.g., mild disabilities). Further studies should also explore the pathogenesis of ANE, including interactions between viral infection and host immune response, as well as the modulatory role of genetic factors.

In conclusion, early identification, timely intervention, and rational treatment are essential to improving outcomes of children with ANE. Clinicians should maintain heightened vigilance during influenza season for patients with prodromal infection rapidly progressing to acute encephalopathy, promptly perform diagnostic evaluations, and initiate corticosteroid therapy within the ultra-early window (≤24 h). Individualized, comprehensive treatment plans, coupled with rehabilitation strategies, can enhance survival and quality of life. Future research should involve larger, multicenter, prospective studies to further elucidate ANE pathogenesis and develop more effective therapeutic strategies, providing a stronger evidence base for prevention and management.

## Data Availability

The raw data supporting the conclusions of this article will be made available by the authors, without undue reservation.
